# Electronic health record-based readmission risk model performance for patients undergoing outpatient parenteral antibiotic therapy (OPAT)

**DOI:** 10.1371/journal.pdig.0000323

**Published:** 2023-08-02

**Authors:** Richard Drew, Ethan Brenneman, Jason Funaro, Hui-Jie Lee, Michael Yarrington, Kristen Dicks, David Gallagher

**Affiliations:** 1 Duke University School of Medicine (Division of Infectious Diseases), Durham, North Carolina, United States of America; 2 Campbell University College of Pharmacy & Health Sciences, Buies Creek, North Carolina, United States of America; 3 Duke University Hospital (Department of Pharmacy), Durham, North Carolina, United States of America; 4 Duke University Biostatistics and Bioinformatics, Durham, North Carolina, United States of America; 5 Duke University Hospital (General Internal Medicine), Durham, North Carolina, United States of America; Massachusetts General Hospital, UNITED STATES

## Abstract

**Background:**

Outpatient Parenteral Antibiotic Therapy (OPAT) provides coordinated services to deliver parenteral antibiotics outside of the acute care setting. However, the reduction in monitoring and supervision may impact the risks of readmission to the hospital. While identifying those at greatest risk of hospital readmission through use of computer decision support systems could aid in its prevention, validation of such tools in this patient population is lacking.

**Objective:**

The primary aim of this study is to determine the ability of the electronic health record-embedded EPIC Unplanned Readmission Model 1 to predict all-cause 30-day hospital unplanned readmissions in discharged patients receiving OPAT through the Duke University Heath System (DUHS) OPAT program. We then explored the impact of OPAT-specific variables on model performance.

**Methods:**

This retrospective cohort study included patients ≥ 18 years of age discharged to home or skilled nursing facility between July 1, 2019 –February 1, 2020 with OPAT care initiated inpatient and coordinated by the DUHS OPAT program and with at least one Epic readmission score during the index hospitalization. Those with a planned duration of OPAT < 7 days, receiving OPAT administered in a long-term acute care facility (LTAC), or ongoing renal replacement therapy were excluded. The relationship between the primary outcome (unplanned readmission during 30-day post-index discharge) and Epic readmission scores during the index admission (discharge and maximum) was examined using multivariable logistic regression models adjusted for additional predictors. The performance of the models was assessed with the scaled Brier score for overall model performance, the area under the receiver operating characteristics curve (C-index) for discrimination ability, calibration plot for calibration, and Hosmer-Lemeshow goodness-of-fit test for model fit.

**Results:**

The models incorporating maximum or discharge Epic readmission scores showed poor discrimination ability (C-index 0.51, 95% CI 0.45 to 0.58 for both models) in predicting 30-day unplanned readmission in the Duke OPAT cohort. Incorporating additional OPAT-specific variables did not improve the discrimination ability (C-index 0.55, 95% CI 0.49 to 0.62 for the max score; 0.56, 95% CI 0.49 to 0.62 for the discharge score). Although models for predicting 30-day unplanned OPAT-related readmission performed slightly better, discrimination ability was still poor (C-index 0.54, 95% CI 0.45 to 0.62 for both models).

**Conclusion:**

EPIC Unplanned Readmission Model 1 scores were not useful in predicting either all-cause or OPAT-related 30-day unplanned readmission in the DUHS OPAT cohort. Further research is required to assess other predictors that can distinguish patients with higher risks of 30-day unplanned readmission in the DUHS OPAT patients.

## Author summary

Administration of parenteral antibiotics outside an institution-based setting is greatly facilitated by structured multidisciplinary programs known as Outpatient Antibiotic Therapy (OPAT). However, up to 25% of OPAT patients require hospital readmission within 30 days. This highlights the need for decision support tools that would facilitate the identification of patients at the highest risk of hospital readmission. One such tool widely available and evaluated in various patient cohorts is the EPIC Unplanned Readmission Model. However, its ability to predict hospital readmission among OPAT patients has not been previously reported. This retrospective cohort study included adult patients discharged with OPAT care with at least one Epic readmission score during the index hospitalization. We found the model was not useful in predicting either all-cause or OPAT-related 30-day unplanned readmission. Our study highlights the need for further research to identify data tools that can identify the OPAT patients at the highest risk of increased healthcare utilization.

## Introduction

Outpatient Antibiotic Therapy (OPAT) is a structured program that coordinates the administration of parenteral antibiotics outside of the acute care setting. The OPAT program at our institution was established in 2016, and has grown to include a core group of pharmacists, nurses, and infectious diseases providers. OPAT nurses and pharmacists review weekly laboratory work and adjust medications. OPAT patients are seen by infectious diseases providers during their OPAT course. The intended result is to save hospital resources and alleviate patient inconvenience and cost. However, there are risks of increased treatment-related adverse effects and hospital readmission due to reduction in direct patient monitoring [1,2].

The U. S. Department of Health and Human Services Agency for Healthcare Research and Quality utilizes measurements of 30-day all-cause readmission as a metric reflecting patient harm [3]. In OPAT patients, reported rates of these readmissions have ranged from 20–26% [4–8]. Improved OPAT patient selection or closer outpatient follow-up have the potential to improving treatment outcomes while minimizing costs to the healthcare system, especially when applied to those at greatest risk for unplanned hospital admissions. Predictive models (including electronic health record-based clinical decision support systems) could aid in identifying such patients. Ideally, such a system would utilize factors known and up-to-date at the time of discharge. One such tool is EPIC’s Risk of Unplanned Readmission Model, a decision support tool of the Epic Electronic Medical Record in use for reducing readmissions at DUHS since 2017. The utility of the model has previously been investigated on our DUHS inpatient population [9,10]. While such a tool has been evaluated for their ability to predict 30-day unplanned readmission at the time of discharge [10,11], evidence regarding its application in the OPAT population is lacking.

We sought to determine the ability of the electronic health record-embedded EPIC Unplanned Readmission Model 1 to predict all-cause 30-day hospital unplanned readmissions in discharged patients receiving OPAT through the DUHS OPAT program. We then evaluated the impact on the Epic model’s discriminatory ability of the DUHS-specific predictors individually on the Epic model’s discriminatory ability on 30-day unplanned readmissions, as well as the discriminatory ability of the Epic readmission score to predict 30-day OPAT-related unplanned readmissions.

## Materials and methods

This retrospective cohort study (reviewed and exempted by the Duke University Hospital Institutional Review Board) was a secondary analysis of data obtained for the evaluation of a published predictive tool for the determination of risk for unplanned hospital readmission in OPAT patients within our academic, tertiary care hospital system [6]. Patients were included if they were Duke University Health System (DUHS) inpatients ≥ 18 years of age enrolled in the DUHS OPAT program (OPAT care initiated inpatient and coordinated by the DUHS OPAT program) discharged to a home or skilled nursing facility between July 1, 2019 –February 1, 2020 and with a planned duration of OPAT at hospital discharge ≥ 7 days, and with an Epic readmission score available during the index hospitalization. Patient populations not routinely cared for by the OPAT service (patients with solid organ and hematopoietic stem cell transplants patients, and those with cystic fibrosis, or left ventricular assist device (LVAD), receiving OPAT administered in a long-term acute care facility (LTAC), or ongoing renal replacement therapy) and those lost to follow-up were excluded.

Patients for study eligibility screening were identified from the OPAT Pharmacist Patient Care List. Data extracted from Duke Maestro Care using the Duke Enterprise Data Unified Content Explorer (DEDUCE) included patient demographics, infection-, treatment- and hospitalization-related data (including dates and indication for index and readmissions within 30 days), select comorbidities (in order to determine the Charlson Comorbidity Index Score [12]), infectious diseases outpatient follow-up, and management of any treatment-related adverse events. A manual chart review was conducted to collect antibiotics and treatment outcomes data. Patients were followed until the end of the OPAT service, or 30 days after the index discharge, or January 3, 2022, whichever occurred later. Data were stored in REDCap (Research Electronic Data Capture), a secure, web-based software platform electronic data capture tool hosted at Duke University Hospital [13].

The primary outcome was the 30-day unplanned readmission, defined as any unplanned hospital readmission to a DUHS hospital for any reason within 30 days of discharge from the index OPAT admission. Readmission was at the discretion of the admitting clinician based on indication. For the secondary analysis, we defined the 30-day OPAT-related unplanned readmission as any readmission due to failure or adverse events associated with OPAT or its administration within 30 days of discharge from the initial OPAT admission. The association of the event to OPAT was determined by one of the investigators (EB) after a review of the electronic health record. For patients with multiple OPAT episodes, we performed individual-level analysis by randomly selecting one encounter per patient.

The primary predictors were the discharge and maximum Epic readmission scores during the index admission. The Epic Unplanned Readmission Risk Model (version 1) risk model variables include patient age, clinical diagnoses, laboratory values, medication numbers and classes, order types, and healthcare utilization variables. These variables are then weighted (using Epic proprietary calculations) to create the overall risk model score. The risk model calculates a score every 4 hours for the readmission risk for inpatients. The score is a continuous variable from 0 to 100 which increases with readmission risk but does not assign a specific probability risk. The score is available to clinical inpatient teams on their patient lists so they can implement readmission risk reduction strategies for patients at high risk for unplanned readmissions. For purposes of this study, the Epic readmission score was calculated daily during the index admission. The last Epic readmission score before discharge was the discharge score. The maximum EPIC readmission score during the index admission was the maximum score, which captures the most severe situations of the hospitalization and has been used in previously published literature [10,11,14]. Additional predictors we assessed included patient age at index admission, vancomycin use, intravenous drug abuse, and OPAT-delivered mode (skilled nursing facility or home service).

We fit separate univariable logistic regression models using 30-day unplanned readmission or 30-day unplanned OPAT-related readmission as the outcomes, and discharge or max Epic readmission scores as the primary predictor. Multivariable logistic regression models were later fit by adjusting for additional DUHS-specific predictors. Unadjusted and adjusted odds ratios (ORs) of the predictors were reported with 95% confidence intervals (CIs). The performance of the models was assessed with the scaled Brier score for overall model performance, the area under the receiver operating characteristics curve (C-index) for discrimination ability, calibration plot for calibration, and Hosmer-Lemeshow goodness-of-fit test for model fit. The scaled Brier score can range from minus infinity to 1. A negative scaled Brier score means the forecast is less accurate than predicting using the average probability of the outcome. A scaled Brier score of 0 indicates it performs the same as predicting using the average probability of the outcome, whereas a score close to 1 indicates it performs much better than using the average probability of the outcome. All statistical analyses were performed by SAS 9.4 (SAS Institute, Cary, NC, USA) and R 4.1.3 (R Core Team, Vienna, Austria).

## Results

From the 606 distinct OPAT encounters that were identified, 115 episodes were excluded for not meeting eligibility criteria. After randomization and removal of an additional 24 episodes for multiple OPAT encounters with the same patient, 467 unique encounters (representing 467 unique patients) were included in the analysis.

Demographic and clinical characteristics of the study population with and without 30-day unplanned readmission are summarized in **Table 1**. Overall, the population consisted predominately of males (60.2%), White race (70.4%) with a median age of 62 (interquartile range [IQR] 52, 72) years. The predominant infection site was bone and joint (58.7%). The duration of treatment was a median of 33 (IQR 18.5, 38) days. Intravenous vancomycin (36.2%) and intravenous cephalosporins (54.6%) were the predominant treatments.

**Table 1:** Demographic and clinical characteristics by 30-day unplanned (all-cause) readmission.

	No readmission	Unplanned readmission	Total
	N = 373	N = 94	N = 467
**Age at Index Admission (y)**			
Median (IQR)	63 (53, 73)	58 (47, 71.8)	62 (52, 72)
Range	(19, 97)	(18, 95)	(18, 97)
**Sex**			
Female	148 (39.7%)	38 (40.4%)	186 (39.8%)
Male	225 (60.3%)	56 (59.6%)	281 (60.2%)
**Race**			
2 or more races	2 (0.5%)	0 (0.0%)	2 (0.4%)
American Indian or Alaskan Native	3 (0.8%)	2 (2.1%)	5 (1.1%)
Asian	4 (1.1%)	2 (2.1%)	6 (1.3%)
Black or African American	83 (22.3%)	29 (30.9%)	112 (24.0%)
White	269 (72.1%)	60 (63.8%)	329 (70.4%)
Not Reported/Declined	6 (1.6%)	1 (1.1%)	7 (1.5%)
Other	6 (1.6%)	0 (0.0%)	6 (1.3%)
**Discharge Service**			
(unspecified)	0	1	1
Cardiology	20	5	25
Cardiothoracic Surgery	18	6	24
General Medicine	99	26	125
General Surgery	20	4	24
General/Thoracic/Cardiothoracic Surgery	11	2	13
Hematology/Oncology	2	0	2
Mother/Baby Care	1	0	1
Neurology / Neurosurgery	7	4	11
Neurology Oncology	10	2	12
Neurosurgery	17	4	21
Obstetrics/Post-partum	1	1	2
Oncology	11	4	15
Orthopedic Surgery	71	12	83
Renal/Pulmonary/MICU	3	2	5
Surgery General/Bariatric	4	0	4
Surgery Orthopedics/Neurology	36	6	42
Telemetry	4	2	6
Urology/Otorhinolaryongology/Ophthalmonlogy/Plastics/Gyenecologic Surgery	38	13	51
**Select Comorbidities**			
myocardial infarction	46 (12.3%)	13 (13.8%)	59 (12.6%)
congestive heart failure	99 (26.5%)	20 (21.3%)	119 (25.5%)
peripheral vascular disease	105 (28.2%)	20 (21.3%)	125 (26.8%)
cerebrovascular disease	63 (16.9%)	15 (16.0%)	78 (16.7%)
dementia	39 (10.5%)	10 (10.6%)	49 (10.5%)
chronic pulmonary disease	109 (29.2%)	26 (27.7%)	135 (28.9%)
rheumatic disease	28 (7.5%)	8 (8.5%)	36 (7.7%)
peptic ulcer disease	13 (3.5%)	1 (1.1%)	14 (3.0%)
diabetes without chronic complications	41 (11.0%)	7 (7.4%)	48 (10.3%)
diabetes with chronic complications	90 (24.1%)	25 (26.6%)	115 (24.6%)
renal disease, mild to moderate	88 (23.6%)	28 (29.8%)	116 (24.8%)
renal disease, severe	10 (2.7%)	6 (6.4%)	16 (3.4%)
hemiplegia or paraplegia	26 (7.0%)	12 (12.8%)	38 (8.1%)
any malignancy	55 (14.7%)	12 (12.8%)	67 (14.3%)
metastatic solid tumor	26 (7.0%)	8 (8.5%)	34 (7.3%)
liver disease, mild	41 (11.0%)	14 (14.9%)	55 (11.8%)
liver disease, moderate to severe	11 (2.9%)	3 (3.2%)	14 (3.0%)
HIV infections, no AIDS	4 (1.1%)	0 (0.0%)	4 (0.9%)
AIDS	2 (0.5%)	2 (2.1%)	4 (0.9%)
IV Drug Abuse	19 (5.1%)	5 (5.3%)	24 (5.1%)
**Charlson Comorbidity Score**			
Mean (SD)	3.4 (2.7)	3.7 (2.9)	3.4 (2.8)
Median (IQR)	3 (1, 5)	3 (2, 5)	3 (1, 5)
Range	(0, 14)	(0, 13)	(0, 14)
**Indication of OPAT**			
Bone or joint infection	220 (59.0%)	54 (57.4%)	274 (58.7%)
Endovascular infection	49 (13.1%)	15 (16.0%)	64 (13.7%)
Skin and soft tissue infection	26 (7.0%)	7 (7.4%)	33 (7.1%)
Respiratory disease	13 (3.5%)	2 (2.1%)	15 (3.2%)
Urogenital infection	17 (4.6%)	5 (5.3%)	22 (4.7%)
Other indication	48 (12.9%)	11 (11.7%)	59 (12.6%)
**Initial Location of OPAT administration**			
Home (Self/Caregiver)	262 (70.2%)	70 (74.5%)	332 (71.1%)
Skilled Nursing Facility	111 (29.8%)	24 (25.5%)	135 (28.9%)
**IV access**			
PICC	351 (94.1%)	85 (90.4%)	436 (93.4%)
Midline CVC	2 (0.5%)	2 (2.1%)	4 (0.9%)
Tunneled CVC	12 (3.2%)	4 (4.3%)	16 (3.4%)
Port	7 (1.9%)	3 (3.2%)	10 (2.1%)
Other	1 (0.3%)	0 (0.0%)	1 (0.2%)
**OPAT antibiotic description**			
aminoglycosides	1 (0.3%)	0 (0.0%)	1 (0.2%)
carbapenems	52 (13.9%)	12 (12.8%)	64 (13.7%)
cephalosporins-oral	35 (9.4%)	7 (7.4%)	42 (9.0%)
cephalosporins-IV	196 (52.5%)	59 (62.8%)	255 (54.6%)
penicillins-oral	26 (7.0%)	4 (4.3%)	30 (6.4%)
penicillins-IV	40 (10.7%)	13 (13.8%)	53 (11.3%)
vancomycin-oral	6 (1.6%)	1 (1.1%)	7 (1.5%)
vancomycin-IV	140 (37.5%)	29 (30.9%)	169 (36.2%)
other oral antibiotics	145 (38.9%)	36 (38.3%)	181 (38.8%)
other intravenous antibiotics	35 (9.4%)	15 (16.0%)	50 (10.7%)
**Number of Hospitalizations in prior 12 months**			
Mean (SD)	0.9 (1.3)	0.9 (1.2)	0.9 (1.3)
Median (IQR)	0 (0, 1)	1 (0, 1)	0 (0, 1)
Range	(0, 7)	(0, 6)	(0, 7)
**Duration of OPAT (days)**			
Mean (SD)	30.9 (17.5)	26.3 (20.1)	30 (18.1)
Median (IQR)	35 (21, 38)	24 (10, 37.8)	33 (18.5, 38)
Range	(7, 239)	(1, 122)	(1, 239)

AIDS, acquired immunodeficiency syndrome; CVC, central venous catheter; HIV, human immunodeficiency virus; IQR, interquartile range; IV, intravenous; MICU, medical intensive care unit; OB, obstetrics; OPAT, outpatient parenteral antibiotic therapy; PICC, peripherally-inserted central catheter; SD, standard deviation

EPIC scores in patients with and without 30-day unplanned readmission are summarized in **Table 2**. The median number of scores was 7 (IQR 5, 12). The mean of the maximum and discharge Epic scores were 18.2 (standard deviation [SD] 9) and 16.9 (SD 8.5), respectively.

**Table 2:** Summary of Epic risk scores by 30-day unplanned (all cause) readmission.

	No Readmission	Unplanned Readmission	All Patients	
	N = 373	N = 94	N = 467	P- value
**Number of Epic risk scores during the index admission**				
Mean (SD)	9.6 (6.9)	9.2 (6.8)	9.5 (6.9)	
Median (IQR)	7 (5, 12)	7 (5, 11)	7 (5, 12)	
Range	(1, 44)	(2, 40)	(1, 44)	
**Maximum Epic risk scores during the index admission**				
Mean (SD)	18.2 (9)	18.5 (9.1)	18.2 (9)	**0.78**
Median (IQR)	17 (11, 22)	17 (12, 22)	17 (11.5, 22)	
Range	(4, 55)	(7, 67)	(4, 67)	
**Epic risk scores at index discharge**				
Mean (SD)	16.8 (8.4)	17.2 (8.7)	16.9 (8.5)	**0.69**
Median (IQR)	15 (11, 21)	15 (11, 21.8)	15 (11, 21)	
Range	(4, 55)	(6, 62)	(4, 62)	

IQR, interquartile range; S.D., standard deviation; p-values were from two-sample t-tests.

**Table 3** summarizes the admission-related clinical outcomes. Of the 467 unique admissions, 94 (20.1%) encountered unplanned readmission during the 30-days post-discharge, and 56 (12%) were OPAT-related. Among the 56 unplanned OPAT-related admissions, infection-related and antibiotic-related adverse effects accounted for 30 (53.6%) and 17 (30.4%), respectively.

**Table 3:** Readmissions during 30-day post-index.

	Total
	N = 467
Readmission	
Any readmission	105 (22.5%)
Planned readmission*	13 (2.8%)
Unplanned readmission*	94 (20.1%)
Time from discharge to first unplanned readmission (day)	
Mean (SD)	12.1 (8)
Median (IQR)	12 (5, 17)
Range	(1, 30)
Unplanned OPAT-related readmission	56 (12.0%)
Infection-related adverse effect	30 (6.4%)
Antibiotic-related adverse effect	17 (3.6%)
IV access	2 (0.4%)
Other	7 (1.5%)

IQR, interquartile range; S.D., standard deviation. *Because some patients had both 30-day planned and unplanned readmission, the sum of the two is larger than “any readmission”.

**Fig 1:**
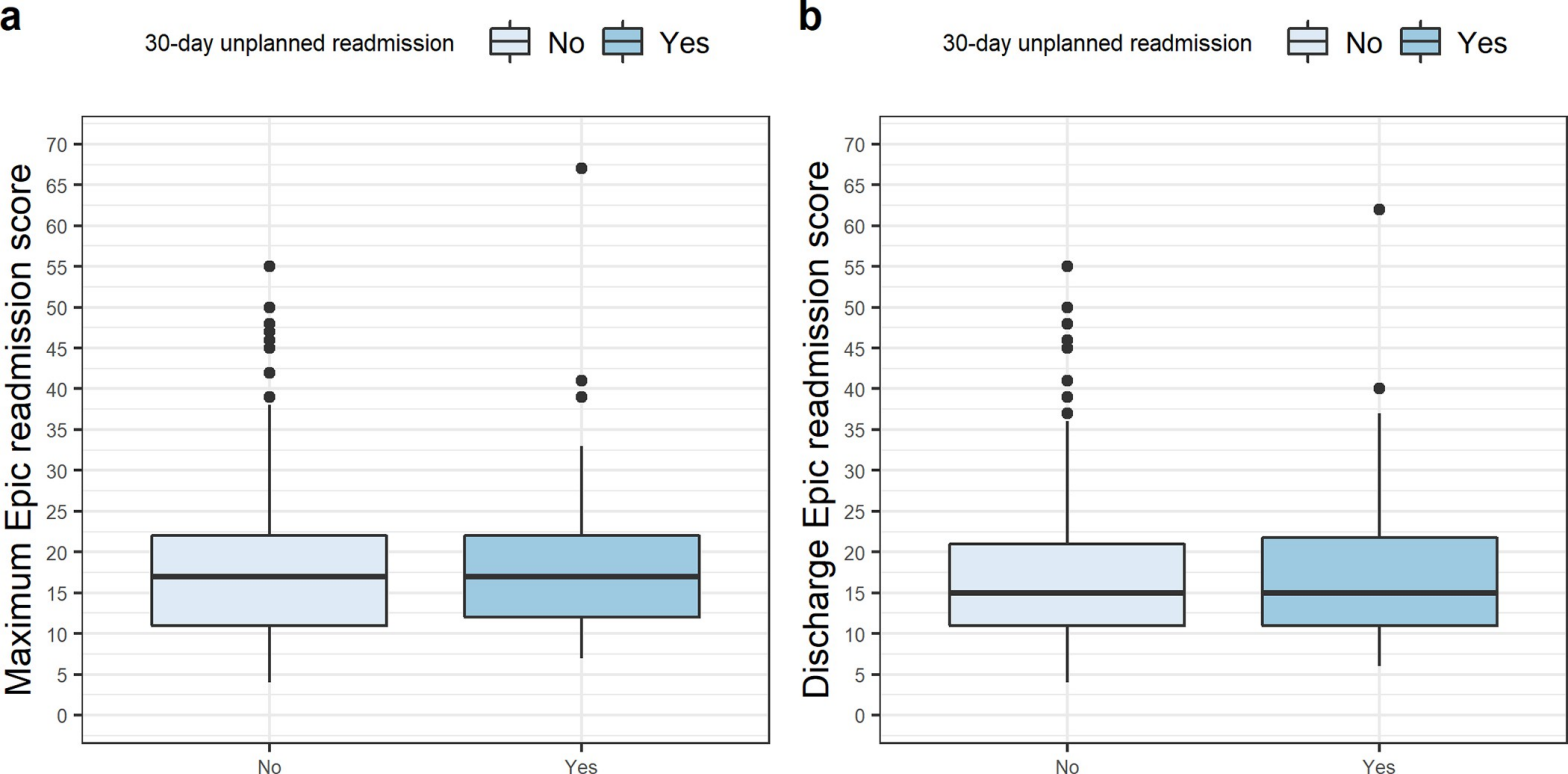
(a) Boxplot of maximum Epic readmission scores during the index hospitalization by 30-day unplanned readmission. (b) Boxplot of discharge Epic readmission scores during the index hospitalization by 30-day unplanned readmission.

**Fig 1.** shows the boxplots of the maximum and discharge readmission scores by 30-day unplanned readmission, respectively. From univariable logistic regression, neither the maximum Epic risk score nor the discharge Epic risk score was associated with 30-day unplanned readmission (unadjusted OR 1.00, 95% CI 0.98 to 1.03; and 1.01, 95% CI 0.98 to 1.03, respectively). **Table 4** summarizes the multivariable logistic regression for 30-day unplanned readmission, using the maximum and discharge Epic readmission scores as the primary predictor. None of the predictors included (patient age at index admission, vancomycin use, IV drug abuse, and OPAT delivered mode [skilled nursing facility or home service]) was associated with 30-day unplanned readmission.

**Table 4:** Multivariable logistic regression to assess the association with 30-day unplanned readmission using the maximum and discharge Epic readmission score as the primary predictor.

Covariates	Levels	Maximum EPIC score Adjusted OR (95% CI)	Discharge EPIC score Adjusted OR (95% CI)
Epic risk score		1.01 (0.98–1.03, p = 0.53)	1.01 (0.98–1.04, p = 0.45)
Age (1-year increase)		0.99 (0.98–1.01, p = 0.25)	0.99 (0.98–1.01, p = 0.24)
Vancomycin use before index discharge	No	Reference	Reference
	Yes	0.74 (0.45–1.20, p = 0.23)	0.74 (0.45–1.20, p = 0.23)
Intravenous drug abuse	No	Reference	Reference
	Yes	0.87 (0.28–2.33, p = 0.80)	0.88 (0.28–2.34, p = 0.81)
Mode of OPAT delivery	Home	Reference	Reference
	Skilled Nursing Facility	0.86 (0.48–1.50, p = 0.61)	0.86 (0.48–1.49, p = 0.59)

The performance of the four Epic readmission score models for predicting 30-day unplanned readmission are summarized in **Table 5**. The C-indices were all very close to 0.5, indicating poor discrimination ability. Adding other predictors only slightly improved performance. The Hosmer-Lemeshow tests were all above 0.05, suggesting no evidence of the lack of fit of the models. We also observed zero or close to zero scaled Brier scores, indicating the forecast was not more accurate than predicting using the average probability of the outcome.

**Table 5:** Model performance of the four Epic readmission score models for predicting 30-day unplanned readmission.

Methods	Maximum Epic readmission score	Discharge Epic readmission score	Maximum Epic readmission score with additional variables	Discharge Epic readmission score with additional variables
Discrimination, c-index (95% CI)	0.51 (0.45, 0.58)	0.51 (0.45, 0.58)	0.55 (0.49, 0.62)	0.56 (0.49, 0.62)
Hosmer-Lemeshow (df)	5.37 (8)	8.91 (8)	10.46 (8)	10.46 (8)
Hosmer-Lemeshow p-value	0.718	0.35	0.234	0.234
Scaled Brier score	0	0	0.01	0.01

**Fig 2:**
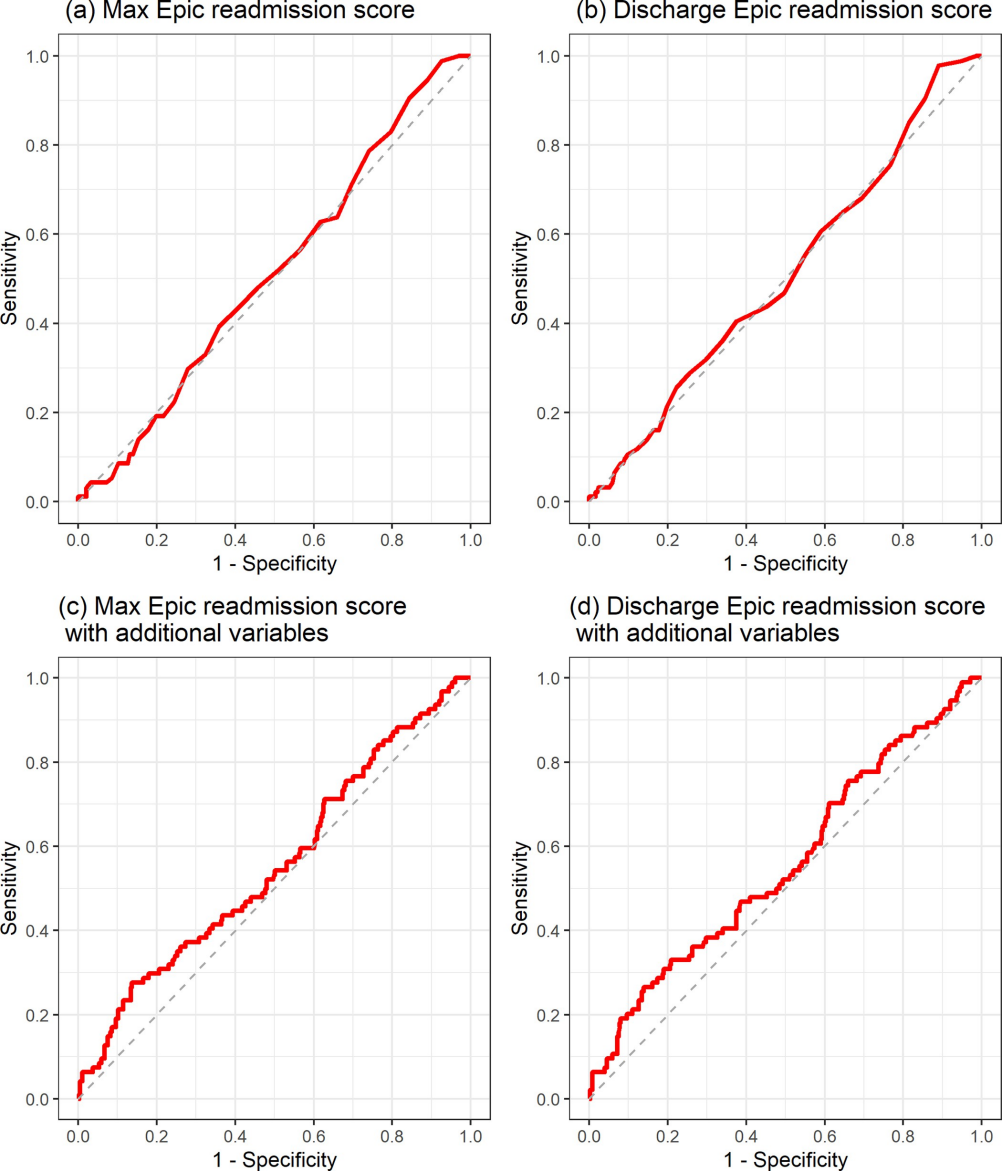
Receiver operating curves (ROCs) of the four models utilizing Epic readmission scores to predict 30-day unplanned readmission.

**Fig 3:**
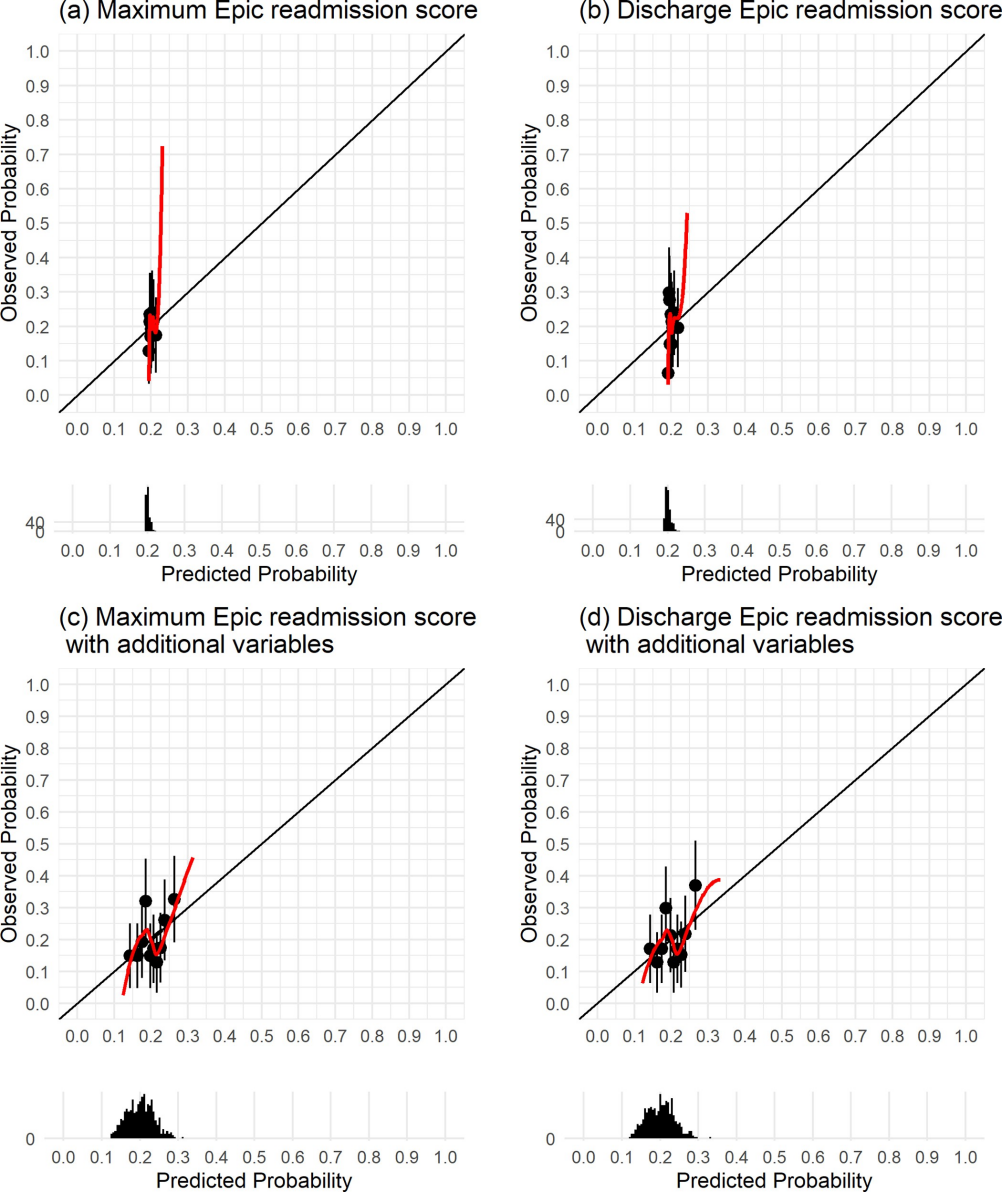
Calibration plots of the four models utilizing Epic readmission scores to predict 30-day unplanned readmission.

Receiver operating curves (ROCs) of the four models utilizing Epic readmission scores to predict 30-day unplanned readmission are provided in **Fig 2**, which showed no discrimination ability among any of the models. Calibration plots of the four models utilizing Epic readmission scores to predict 30-day unplanned readmission are provided in **Fig 3,** which showed poor agreement between the predicted (from the models) and observed numbers of unplanned readmission.

## Discussion

In contrast to previous reports evaluating the utility of the EPIC Unplanned Readmission Model 1 in predicting 30-day unplanned hospital readmissions conducted within DUHS on a variety of clinical service cohorts, we found poor discrimination ability among models utilizing maximum and discharge Epic readmission scores in predicting both all-cause and OPAT-related 30-day unplanned readmission in the DUHS OPAT cohort [10,11]. We believe several factors may influence such differences, including the method in which the score is calculated, performance variability in different patient cohorts, and differences in study endpoints.

While the present study included one score per day, the Epic Readmission Risk Model V.1 calculates a readmission risk score every 4 hours for the hospitals within DUHS. The model variables include patient age, clinical diagnoses, laboratory values, medication numbers and classes, order types, and other healthcare use variables [9,15]. The resulting scores are provided as a continuous variable (from 0 to 100). Previous studies have indicated that higher values represent increasing risk [10,11]. However, the significance of each Epic readmission score data point is unknown, as the score can fluctuate based on a patient’s hospital course. In the present study, the Epic risk score did not change dramatically during the hospitalization for most encounters. The maximum and discharge risk scores only differed by 1.3 points on average. Additionally, the discharge Epic risk score was usually not the lowest score during the hospitalization, but rather 3.5 points higher (on average). Neither maximum nor discharge scores had good model performance in predicting unplanned readmission.

Differences in patient cohorts between studies make comparisons difficult, even within OPAT populations [6]. Such differences likely impact patient characteristics, outcome prevalence, and systematic differences in observations. Prior studies have reported variable performance of the Epic risk score when evaluated in different (non-OPAT) inpatient cohorts [10]. In general, the model appears to have reduced performance in patients with higher risk scores and rates of readmission. For example, general hospital readmission rates during the study period ranged from 10–12% across the three hospitals within the DUHS network where C-index ranged from 0.72–0.76 [10]. However, in the subset Duke University Oncology population, 30-day unplanned readmission rates of 22% demonstrated the lowest C-index (0.611) [10]. Similar findings were observed in another study comparing patients with chronic kidney disease requiring dialysis who have higher rates of readmission when compared to the general patient population [11]. The area under the receiver operating characteristic curve for the readmission risk score was statistically lower for patients receiving dialysis (0.681) versus general patients (0.705) (P < 0.004). In addition to the incidence of readmission being higher than many patient populations, the reasons for readmission in the OPAT population are more diverse (including ongoing treatment-related adverse events, superinfection, and those related to maintaining intravenous access). In the present study, most of our patients would have been otherwise classified in both the General Medicine and Orthopedic Surgery populations (125 and 83 patients, respectively). A higher percentage of our subjects were male and White than reported in prior studies assessing the utility of the score [10,11] and that observed in our general adult medicine population (personal communication, DG). Performance of the Epic score may be lower in surgical patients, since post-operative complications are a significant driver for readmissions and are not captured with the Epic score.

The fixed model and factor weighting may limit the utility of the EPIC model in predicting readmission in selected cohorts. Previous studies evaluating readmission risk models within the OPAT patient population have used prior hospitalizations in the preceding 12 months, concurrent IV antimicrobial therapy, type of infection and mode of OPAT treatment [5, 7, 8]. While age and Charlson scores were predictive in some studies [5], they were less helpful in others [7]. In addition, socioeconomic factors may be less impactful in the OPAT population likely due to OPAT eligibility screening [1,16]. Discharge to a skilled nursing facility or subacute rehabilitation center was found to be a risk factor [8,17]. Infectious diseases (ID) service follow-up has been associated with decreasing risks. In one study, patients without such follow-up experienced an increase in both catheter-related bloodstream infections (OR, 3.78; P = .007) and 30-day readmission (OR, 2.59; P < .001) [17]. In another report, ID outpatient follow-up within 2 weeks was associated with a lower risk of all-cause 30-day readmission (adjusted odds ratio, 0.33; P = .0001). [18] In the present study, we observed a statistically significant difference in the proportions of patients with follow-up with infectious disease clinics after OPAT between those with and without 30-day unplanned readmission (p<0.001). Specifically, 60.6% of the patients with 30-day unplanned readmission had no follow-up with ID, whereas only 23.3% of the patients without 30-day unplanned readmission had no follow-up with ID. However, patients could have been readmitted and thus follow-up visits with ID were canceled. Because we cannot separate the causal relationship between follow-up with infectious disease clinics and unplanned readmission, this predictor was not included in the regression model. Our efforts to introduce additional OPAT-specific factors (age, vancomycin use before index discharge, IV drug abuse, and mode of OPAT delivery in a skilled nursing facility) did not improve the discrimination ability of the tool.

Other differences exist between the present study and prior reports. In the prior studies, the percentage of patients receiving OPAT was not specifically reported. Rates of 30- day hospital readmission may further be characterized by cause (“all-cause” versus exposure-related, such as OPAT) and as planned or unplanned. Similar to several other studies and consistent with the application of the score, we first sought to characterize 30-day (all-cause) unplanned readmission rates. Others have reported 7, 14, and 30 days and that the utility of the model declined after 7 days post-discharge [14]. Unplanned readmissions within 30 days have also been utilized [15]. In addition to the timing, such endpoints capture both planned readmissions, as well as admissions unrelated to the target intervention. We examined the ability of the model to predict both all-cause and OPAT-related 30-day readmissions, but the model failed to predict either event.

There are limitations to our evaluation worth discussion. The single-center nature of the study may limit generalizability to institutions without comparable OPAT populations. As previously discussed, we limited our characterization of the EPIC score to discharge and maximum Epic readmission scores in the predictive models. There could be other important predictors for unplanned readmission that we did not include in the study, such as service line (general medicine vs. orthopedics vs. cardiology vs. trauma/surgery). While hospital readmission data was limited to hospitals within the DUHS system, we feel the close patient follow-up provided by the OPAT service would have detected the unlikely admission to other healthcare facilities. Attribution of the readmission reason as related or unrelated to OPAT was made by the investigators. Since we intentionally chose the timing of the cohort selection to provide data free of the potential influence of the COVID19 pandemic on hospital admission and readmission rates, it is unknown whether such findings would be impacted by such extraordinary circumstances. Other investigators have reported EHR-based tools to predict hospital readmission risk in patients admitted due to COVID19 infection with similar or superior performance characteristics [19].

## Conclusions

The models incorporating maximum or discharge Epic readmission scores showed poor discrimination ability in predicting 30-day unplanned readmission in the DUHS OPAT cohort. Incorporating additional DUHS-specific variables, including age, vancomycin use before index discharge, IV drug abuse, and mode of OPAT delivery in a skilled nursing facility did not improve the discrimination ability. The models for predicting 30-day unplanned OPAT-related readmission performed slightly better but the discrimination ability was still poor. There remains something unique in the OPAT population influencing readmissions that is not captured in the standard readmission risk model (such as the Epic model) despite our attempts to improve the model’s discriminating ability by adding variables identified by other investigators. This may include factors such as such as the patients’ subjective needs and/or cost-of-care reimbursement. We believe that more research needs to be done to investigate what unique socioeconomic or clinical factors may be responsible for readmissions in the OPAT population.
